# Antibacterial activity of an acidic phospholipase A_2_ (NN-XIb-PLA_2_) from the venom of *Naja naja* (Indian cobra)

**DOI:** 10.1186/s40064-016-1690-y

**Published:** 2016-02-03

**Authors:** S. Sudarshan, B. L. Dhananjaya

**Affiliations:** Venom Research Unit, Adichunchanagiri Biotechnology and Cancer Research Institute (ABCRI), Balagangadharanatha Nagara, Mandya District, Mandya, Karnataka 571 448 India; Toxinology/Toxicology and Drug Discovery Unit, Center for Emerging Technologies, Jain University, Jain Global Campus, Kanakapura Taluk, Ramanagara, Karnataka 562112 India

**Keywords:** Snake venom, Phospholipase A_2_, Antibacterial, *Naja naja*, Human pathogenic bacteria

## Abstract

The resistance of bacteria against the use of conventional antibiotics has become a serious threat to public health and considering the associated side effect with antibiotics; new strategies to find and develop new molecules with novel modes of action has received grate attention in recent years. In this study, when the antibacterial potential of an acidic protein—NN-XIb-PLA_2_ (*Naja naja* venom phospholipase A_2_ fraction—XIb) of *Naja naja* venom was evaluated, it showed significant bactericidal action against the human pathogenic strains tested. It inhibited more effectively the gram positive bacteria like *Staphylococcus aureus and Bacillus subtilis*, when compared to gram negative bacteria like *Escherichia coli*, *Vibrio cholerae*, *Klebsiell pneumoniae* and *Salmonella paratyphi*. It inhibited the bacterial growth, with a MIC values ranging from 17 to 20 µg/ml. It was interesting to observe that NN-XIb-PLA_2_ showed comparable antibacterial activity to the used standards antibiotics. It was found that their was a strong correlation between PLA_2_ activities, hemolytic and antibacterial activity. Furthermore, it is found that in the presence of *p*-bromophenacyl bromide (*p*-BPB), there is a significant decrease in enzymatic activity and associated antibacterial activities, suggesting that a strong association exists between catalytic activity and antimicrobial effects, which thereby destabilize the membrane bilayer. These studies encourage further in dept study on molecular mechanisms of bactericidal properties of NN-XIb-PLA_2_ and thereby help in development of this protein into a possible therapeutic lead molecule for treating bacterial infections.

## Background

Worldwide increase in resistance of bacteria for the use of antibiotics and the undesirable side effects associated with it has become a serious public health problem (Norrby et al. 2005; Choudhury et al. 2012; Echols 2012; Ghafur 2013). This resistance to conventional antibiotic has prompted an intensive search for new therapeutic agents from diverse sources, including of animal origin (Zasloff 2002). Proteins/peptides with potent antimicrobial activity from different secretary organisms, include snake (venom) have been identified (Zasloff 2002; Samy et al. 2012). Snake venom, particularly of crotalidae venoms, is a rich source for discovery and development of novel microbicidal agents (Perumal Samy et al. 2006; Samy et al. 2012). Among various components of snake venom, phospoholipase A_2_ (PLA_2_) enzyme apart from the catalytic activity of hydrolyzing the sn-2 ester bond of glycerophospholipids, exhibits diverse biological/pharmacological activities (Kini 1997; Gutiérrez and Lomonte 2013). The diverse arrays of biological actions are either known to be dependent or independent of catalytic activity (Kini 1997; Gutiérrez and Lomonte 2013). svPLA_2_s are also reported as antimicrobial agents and are emphasized for its development into a therapeutic drug for treating infectious diseases (Perumal Samy et al. 2006; Samy et al. 2012). Crotapotin, a secretory phospholipase A_2_ of the *Crotalus durissus terrificus* venom, shows antibacterial activity (Soares et al. 2001a, b) as well as antiviral activity against the human immunodeficiency virus (Toyama et al. 2003; Sampaio et al. 2003). An acidic PLA_2_, both Asp49 and Lys49 PLA2 homologue (Paramo et al. 1998; Vargas et al. 2012), have previously been shown to possess bactericidal activity (Soares et al. 2001a, b). A cationic protein of inland taipan (*Oxyuranus microlepidotus*) venom is demonstrated to selectively and dose-dependently kill gram-positive bacteria through membrane disruption (Nair et al. 2007). Perumal Samy et al. (2010), recently reported a saw-scaled viper venom phospholipase A_2_ with novel bactericidal and membrane damaging activities. Thus svPLA_2_ are demonstrated to be very attractive to be developed as microbicidal therapeutic agents because of their biochemical diversity, broad spectrum of activity against enveloped bacteria, fungi, viruses, protozoa, and parasites (Pereira 2006; Perumal Samy et al. 2006; Samy et al. 2012).

Despite the potential therapeutic application of svPLA_2_s as antimicrobial agents (Pereira 2006; Perumal Samy et al. 2006; Samy et al. 2012), very few svPLA_2_s with microbicidal/antimicrobial activities have been characterized for their mechanism of action (Pereira 2006; Samy et al. 2012; de Oliveira Junior et al. 2013). Indian Cobra (*Naja naja*) species is a widely distributed snake that is responsible for potent toxic and lethal effects (Mukherjee and Maity 2002; Shashidharamurthy et al. 2002, 2010; Dhananjaya et al. 2006; Hiremath et al. 2013). Although several reports exists on its various biological effects (Mukherjee and Maity 2002; Shashidharamurthy et al. 2002, 2010; Dhananjaya et al. 2006; Hiremath et al. 2013). However, there are very limited reports available on the microbicidal activities exhibited by PLA_2_s from Indian Cobra venom (Sudarshan and Dhananjaya 2015). An acidic PLA_2_—NN-XIb-PLA_2_ (*Naja naja* venom phospholipase A_2_ fraction—XIb) isolated from *Naja naja* venom (Rudrammaji and Gowda 1998) is reported for various biological effects (Rudrammaji and Gowda 1998), however studies on its therapeutic properties particulary as an anti-bacterial agent has not been clearly evaluated. Therefore, in this study we evaluate the antibacterial potential of NN-XIb-PLA_2_ and its possible mechanism of action. Further, this study exemplifies the therapeutic utility of NN-XIb-PLA_2_ as an antimicrobial drug/agent.

## Materials and methods

Venom of *Naja naja* was purchased from Irula Co-operative Society Ltd., Chennai, India. Agar, beef extract, yeast extract and peptone were purchased from Hi Media Private Ltd., Mumbai, India. *p*-bromophenacyl bromide (*p*-BPB) and other chemicals used were of all analytical grades purchased from Sigma Chemicals Ltd. USA. Authentic pure clinical isolated cultures of human pathogenic bacteria; *Staphylococcus aureus*, *Bacillus subtilis*, *Escherichia coli*, *Salmonella typhi*, *Vibrio cholerae*, *Klebsiella pneumoniae* and *Salmonella paratyphi* were obtained from the Microbiology Department, Adichunchanagiri Institute of Medical Sciences (AIMS), B.G. Nagara, Karnataka, India. These are all human pathogens that have developed some resistance to common antibiotics particularly in the in the clinical environment. Bacteria were multiplied in nutrient agar at 36 ± 2 °C. After 2 days, cultures were harvested and prepared at a final concentration of 1 × 10^8^ cfu/ml and used for in vitro inhibition assay. All other chemicals used were of analytical grade.

### Isolation of NN-XIb-PLA_2_ and chemical modification by *p*-bromophenacyl bromide

NN-XIb-PLA_2_ from the venom *Naja naja* (Southern region) was purified up to homogeneity as described previously by the method of Rudrammaji and Gowda (1998). The protein concentration was estimated by Lowry’s method. Chemical modification of PLA_2_ by *p*-BPB was carried out as described by Condrea et al. (1981) One hundred microliters of 40 mM *p*-BPB in acetone were added to 3 ml of PLA_2_ solution (0.5 mg/ml, in 0.05 M Tris–HCl buffer, pH 7.5). The reaction was allowed to proceed for 40 min, and then acidified with glacial acetic acid to pH 4.0 to stop the reaction. Excess of reagent was removed by dialysing against 0.05 M Tris–HCl buffer pH 7.5.

### Phospholipase A_2_ activity

The Phospholipase A_2_ assay was carried out according to the method as described by Bhat and Gowda (1989). Phosphatidyl choline was diluted with petroleum ether (60–80 °C) to get a concentration of 1000 nmol/50 ml. The reaction mixture containing NN-XIb-PLA_2_ (6 μg) was made up to 680 ml with water. To the reaction mixture, 200 μl of ether, 100 μl of Tris–HCl buffer (0.05 M, pH 7.5), and 20 μl of CaCl_2_ (0.4 M) was added. The total reaction mixture was incubated at 37 °C for 60 min. After incubation, 0.5 ml of Doles mixture (Isopropanol: Pet ether: 1NH_2_SO_4_, 40:10:1) was added, mixed and centrifuged at 1000 rpm for 3 min. To the organic phase 0.5 ml of CHCl_3_: Pet ether (1:5) was added, mixed and centrifuged at 1000 rpm for 3 min. To the upper phase cobalt reagent [1.35 vol. of Triethanolamine made up to 10 ml with solution A (6 g of CO(NO_3_)2·6H_2_O + 0.8 ml glacial acetic acid) and 7 ml of solution B (Saturated Na_2_SO_4_)] was added, mixed and centrifuged 1000 rpm for 3 min. The upper organic phase was carefully transferred and 0.75 ml of α-nitroso-β-naphthol reagent (0.4 % α-nitroso-β-naphthol in 96 % ethanol) was added. The intensity of the orange colour is directly proportional to the amount of cobalt present. After 30 min 2 ml of ethanol was added to dilute the contents and absorbance was read at 540 nm. The amount of free fatty acid released was estimated using standard linolenic acid curve. The enzyme activity was expressed as nmoles of fatty acid released/min/mg of protein.

For inhibition studies, NN-XIb-PLA_2_ (6 μg) was preincubated with or without different concentration of *p*-BPB (1–6 μm) at 37 °C for 15 min. Appropriate controls were carried and further experiments were carried out as described above. The inhibition is expressed as percentage taking activity of venom alone as 100 %.

### Haemolytic activity assay

Haemolytic (direct/indirect) activity of isolated NN-XIb-PLA_2_ was determined according to the method of Boman and Kaletta (1957), using packed human erythrocytes (blood group A). The direct and indirect haemolytic assays were carried out using washed erythrocytes. For the direct haemolytic assay, packed erythrocytes (1 ml) were suspended in nine volumes of phosphate-buffered saline (PBS), which formed the stock. The stock (1 ml) was incubated with various concentrations of isolated NN-XIb-PLA_2_ (0–5 µg) for 30 min at 37 °C. For the indirect haemolytic assay, stock was prepared by mixing packed erythrocytes (1 ml), egg yolk (1 ml) and phosphate-buffered saline (8 ml). One millilitre of suspension from stock was incubated with various concentrations of isolated NN-XIb-PLA_2_ (0–3 µg) for 30 min at 37 °C. The reaction was terminated by adding 10 ml of ice-cold PBS and then centrifuged at 4 °C and 800 g. The amount of haemoglobin released in the supernatant was measured at 540 nm. One millilitre of stock erythrocytes with 10 ml ice-cold PBS alone was considered as 0 % lysis.

For inhibition studies, NN-XIb-PLA_2_ (3 μg) was preincubated with or without different concentration of *p*-BPB (1–6 μM) at 37 °C for 15 min. Appropriate controls were carried and further experiments were carried out as described above. The inhibition is expressed as percentage taking activity of venom alone as 100 %.

### Bactericidal activity of NN-XIb-PLA_2_

Bactericidal activity was evaluated by the well diffusion method on nutrient agar medium (Forbes et al. 1990). This was confirmed by the inhibitory effect on bacterial growth as reflected by the inhibition zone, compared to that of known antibiotics like Gentamycin (G); Chloramphenicol (Cp) and Streptomycin (Sm) at 30 µg/ml. The sterile nutrient agar medium (20 ml) in petri dishes was uniformly smeared using sterile cotton swabs with test pure cultures of human pathogenic bacteria *S. aureus*, *B. subtilis*, *E. coli*, *S. typhi*, *V. cholerae*, *K. pneumoniae* and *S. paratyphi*. The nutrient agar media was prepared by dissolving 0.3 % beef extract, 0.3 % yeast extract, 0.5 % peptone, 0.5 % NaCl and 1.5 % agar in 1: l of distilled water. The wells of 5 mm diameter were made using a sterile cork borer in each petri dish and the isolated NN-XIb-PLA2 (0–6 µg) pre-incubated independently with or without *p*-BPB (15 μM) were added; a blank well loaded without test compound was regarded as control. For each treatment, 10 replicates were prepared. The plates were incubated at 37 °C for 24 h and the resulting zone of inhibition was measured by comparing control and the standard antibiotics.

For inhibition studies, NN-XIb-PLA_2_ (6 μg) was preincubated with or without different concentration of *p*-BPB (1–6 μM) at 37 °C for 15 min and antimicrobial activity was carried out as described above with appropriate controls. The inhibition is expressed as percentage taking activity of venom alone as 100 %.

### Determination of minimum inhibitory concentration (MIC)

The minimum inhibitory concentration of the isolated NN-XIb-PLA_2_ and the antibiotics used were determined by serial dilution in the nutrient agar, with concentrations ranging from 2 to 20 µg/ml. The inoculum was prepared from fresh overnight broth culture in nutrient broth and plates were incubated for 24 h at 37 °C. MIC was recorded as the lowest NN-XIb-PLA2 and the antibiotics concentration demonstrating no visible growth in the broth (Prescot et al. 1996).

### Statistical analysis

Statistical analysis was done using SPSS (Windows version 10.0.1; SPSS Inc., Chicago, IL) using a one-way student’s *t* test; p < 0.05 was considered as statistically significant, when comparing with relevant controls. All results refer to mean ± SD.

## Results and discussion

**Fig. 1:**
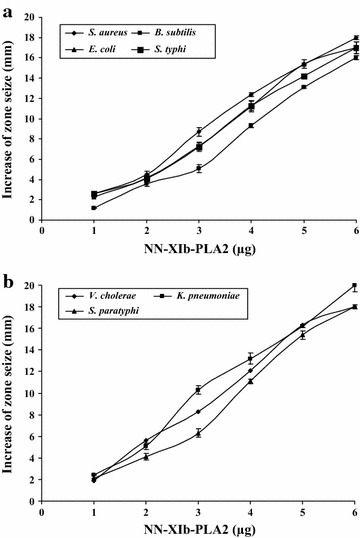
Dose-dependent bactericidal activity of NN-XIb-PLA_2_ **a** bactericidal activity against *S. aureus*, *B. subtiles*, *E. coli*, *S. typhi*, **b** *V. cholerae*, *K. pneumoniae*, *S. paratyphi*. The diameter of the clear zone was measured and plotted after subtracting the diameter of the well (5 mm). Results are mean ± SD for three independent assays each performed in triplicate

**Fig. 2:**
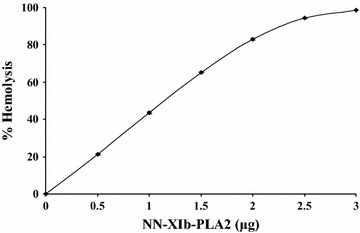
Dose dependent indirect hemolytic activity of NN-XIb-PLA_2_. NN-XIA-PLA_2_ (0–3 µg) in 100 µl of phosphate-buffered Saline (PBS) was incubated with erythrocytes, egg yolk and PBS (1:1:8 v/v) for 10 min at 37 °C. The released hemoglobin in the supernatant was measured by taking absorbance at 540 nm. The results shows ± SEM for n = 4

Snake venom PLA_2_s, apart from their well known diverse biological/pharmacological function are also known to act as antibacterial agents (Samy et al. 2012; de Oliveira Junior et al. 2013). The acidic PLA_2_—NN-XIb-PLA_2_ isolated from *Naja naja* venom (Rudrammaji and Gowda 1998) is reported for various biological effects (Rudrammaji and Gowda 1998). In this study, when evaluated for antibacterial activity on different microbial species, it was observed that NN-XIb-PLA_2_ (0–6 µg/ml) dose-dependently (Fig. 1a, b) had a broad spectrum of very significant antibacterial activity by producing a clear zone of inhibition in the range of 17 ± 2–20 ± 3 mm, which was comparable to the standards used like gentamycin, chlorophenicol and streptomycin (which were in the range of 16–20 mm) (Table 1). When NN-XIb-PLA_2_ was tested, using the agar dilution assay for determining the minimum inhibitory concentration (MIC), it was observed that it inhibited bacterial growth, with MIC values ranging from 19 to 26 µg/ml. It was interesting to observe that NN-XIb-PLA_2_ showed comparable MIC values with standard antibiotics, which ranged from 11.2 to 20 µg/ml (Table 2). Thus, NN-XIb-PLA_2_ is as potent as standard antibiotics in inhibiting the growth of bacterial strains (Fig. 2).

**Table 1:** Antibacterial activity of NN-XIb-PLA_2_ and standard antibiotics

Microorganisms	Diameter of inhibition zone (mm)			
	NN-PL-XIb	G	Cp	Sm
Gram positive				
*Staphylococcus aureus*	18 ± 2	18 ± 1	23 ± 2	26 ± 2
*Bacillus subtilis*	16 ± 3	18 ± 2	19 ± 2	28 ± 3
Gram negative				
*Escherichia coli*	17 ± 2	18 ± 2	18 ± 2	21 ± 2
*Salmonella typhi*	17 ± 3	17 ± 2	18 ± 1	18 ± 1
*Vibrio cholerae*	18 ± 1	16 ± 2	19 ± 2	19 ± 2
*Klebsiella pneumoniae*	20 ± 2	18 ± 2	18 ± 1	21 ± 3
*Salmonella paratyphi*	18 ± 2	19 ± 2	18 ± 2	20 ± 2

The results are mean SD (n = 6)

*G* gentamycin, *Cp* chloramphenicol, *Sm* streptomycin

**Table 2:** Minimum inhibitory concentration (MIC) of NN-XIb-PLA_2_ and antibiotics in serial dilution method

Microorganisms	MIC (µg/ml)			
	NN-PL-XIb	G	Cp	Sm
Gram positive				
*Staphylococcus aureus*	23.3 ± 3	20.8 ± 1	14.4 ± 2	13.6 ± 1
*Bacillus subtilis*	25.1 ± 1	20.8 ± 3	14.4 ± 1	16.6 ± 1
Gram negative				
*Escherichia coli*	19.3 ± 3	23.8 ± 1	14.4 ± 2	14.6 ± 1
*Salmonella typhi*	22.1 ± 3	18.8 ± 1	17.4 ± 2	13.6 ± 1
*Vibrio cholerae*	21.3 ± 2	19.8 ± 3	14.4 ± 1	19.6 ± 1
*Klebsiella pneumoniae*	26.1 ± 3	20.8 ± 1	14.4 ± 2	13.6 ± 1
* Salmonella paratyphi*	21.4 ± 2	23.8 ± 1	14.4 ± 2	14.6 ± 1

The results are mean SD (n = 6)

*G* gentamycin, *Cp* chloramphenicol, *Sm* streptomycin

**Fig. 3:**
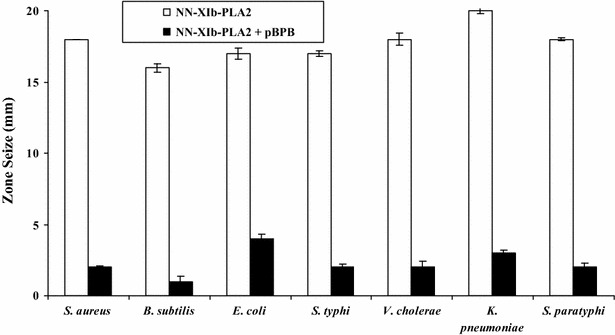
Bactericidal activity against different human pathogenic strains of NN-XIb-PLA_2_. NN-XIb-PLA_2_ (6 μg/ml) was preincubated with or without different concentration of *p*-BPB (6 μM) at 37 °C for 15 min and bactericidal activity was estimated in agar diffusion assay. The diameter of the clear zone was measured and plotted after subtracting the diameter of the well (5 mm). Results are mean ± SD for three independent assays each performed in triplicate

It is usually observed that there is a strong correlation between PLA_2_, hemolytic and antibacterial activities (Samy et al. 2012; de Oliveira Junior et al. 2013; Perumal Samy et al. 2007). When evaluated, it was found that NN-XIb-PLA_2_, dose dependently hemolysed the blood cells and at 3 µg/ml concentration it showed 100 % hemolysis (Fig. 1). From these data it may be concluded that the antibacterial effects of NN-XIb-PLA_2_ is dependent upon catalytic activity i.e. enzymatic membrane degradation effect that is usually observed in svPLA_2_s (Samy et al. 2012; Buckland and Wilton 2000; Sudarshan and Dhananjaya 2015). Also, the correlation between PLA_2_, hemolytic and antibacterial activities, exemplifies that the catalytically activity of PLA_2_ is principally involved in bactericidal/antibacterial activities (Samy et al. 2012; de Oliveira Junior et al. 2013; Perumal Samy et al. 2010; Sudarshan and Dhananjaya 2015), however other mechanism can not be completely ruled out. *Bothrops asper* (also classified within group IIA) snake venom PLA_2_ was shown to directly kill both gram-positive and gram-negative bacteria (Paramo et al. 1998). Further, it was observed that one of the toxin of *B. asper* i.e., myotoxin II, a catalytically-inactive Lys49 PLA_2_ exhibited bactericidal mechanism, independent of its catalytic activity (Paramo et al. 1998; Samy et al. 2012; de Oliveira Junior et al. 2013). Further studies showed that a short sequence of the protein, i.e., corresponding to residues 115–129 of its cytolytic C-terminal region was responsible for its bactericidal activity (Paramo et al. 1998; Samy et al. 2012; de Oliveira Junior et al. 2013), emphasizing the fact that bactericidal activity is not associated with enzymatic activities. In relation to these observation, in our study it was observed that, when the protein was preincubated with *p*-BPB (an inhibitor of svPLA_2_ enzymatic activity) (Rudrammaji et al. 2001), a significant decrease in antibacterial activity was observed (Fig. 3), and complete abolition of antibacterial activity is observed (Table 3), indicating that their was no dissociation of enzymatic activity and bactericidal/antibacterial activity of NN-XIb-PLA_2_. Furthermore, considering the homogenous nature of the protein with no associated other venom enzymatic activities (like L-amino-oxidase, proteases etc..) in the preparation (Data not shown), it may be concluded that the antibacterial activity of NN-XIb-PLA_2_ is dependent upon the catalytic activity i.e. enzymatic membrane degradation effect. However other mechanisms can not be completely ruled out which may include the “fatal depolarization” of the bacterial membrane, creation of physical holes in the membrane, scrambling of normal distribution of lipids between the bilayer leaflets, damage of critical intracellular targets after internalization of the peptide, and also by inhibition of macromolecular biosynthesis as observed in many of svPLA(2)s and/or interacting with specific vital components inside the bacteria (Park et al. 1998; Samy et al. 2012; Sudarshan and Dhananjaya 2015). *Agkistrodon piscivorus piscivorus* PLA_2_s was shown to interact with lipopolysaccharide (LPS) and lipid A from different gram negative bacteria or with the lipoteichoic acid from *Staphylococcus aureus*, and is known to rely on a membrane-permeabilizing mechanism to exert its bactericidal effects (Shen and Cho 1995). Saikia et al. (2012) recently demonstrated that the presence of a large number of PLA_2_-sensitive phospholipid domains/composition, and rather than only the phosphatidylcholine (PC) content of a particular membrane determine the extent of membrane damage by a particular venom PLA_2_ enzyme. As observed in our study this might be one of the reasons of differential inhibitory potency of NN-XIb-PLA_2_ on various bacterial species. However the protein being an acidic PLA_2_ (NN-XIb-PLA_2_) seems to bring out its antimicrobial activity by acting upon the membrane and hydrolyze it through its enzymatic activity. From this study it seems that there is a strong correlation between catalytic activity and antimicrobial effects of NN-XIb-PLA_2_. However, other mechanisms can not be completely ruled out. Therefore, further studies of molecular mechanism of action of NN-XIb-PLA_2_ bactericidal activities will be interesting to develop this as a therapeutic lead molecule for application purpose.

**Table 3:** Antibacterial activity of NN-XIb-PLA_2_ without and with *p*-BPB

Microorganisms	Diameter of inhibition zone (mm)	
	NN-PL-XIb	NN-PL-XIb + *p*-bromophenacyl bromide (*p*-BPB)
Gram positive		
*Staphylococcus aureus*	18 ± 2	02 ± 0.1
*Bacillus subtilis*	16 ± 3	01 ± 0.3
Gram negative		
*Escherichia coli*	17 ± 2	04 ± 0.5
*Salmonella typhi*	17 ± 3	02 ± 0.1
*Vibrio cholerae*	18 ± 1	02 ± 0.4
*Klebsiella pneumoniae*	20 ± 2	03 ± 0.3
*Salmonella paratyphi*	18 ± 2	02 ± 0.3

The results are mean SD (n = 6)

## Conclusion

This study indicates the potential bactericidal activities of NN-XIb-PLA_2_, a PLA_2_ of *Naja naja* venom. A significant decrease in antibacterial activity in presence of *p*-BPB (an inhibitor of PLA_2_ enzymatic activity) was observed, suggesting a correlation between enzymatic and antibacterial activity. Also, it may pocess other properties that mimic the bactericibal/membrane permeability-increasing protein. Thus these studies encourage further in dept study on molecular mechanisms of anti-bacterial properties and thereby help in development of this protein into therapeutic lead molecule for treating bacterial infections.
